# A remarkably modified species of the tribe Platynini (Coleoptera, Carabidae) from a limestone cave in Jiangxi Province, eastern China

**DOI:** 10.3897/zookeys.382.6740

**Published:** 2014-02-19

**Authors:** Jianmei Pang, Mingyi Tian

**Affiliations:** 1 Department of Entomology, College of Natural Resources and Environment, South China Agricultural University, 483 Wushanlu, Guangzhou, Guangdong, 510642, China

**Keywords:** *Morimotoidius*, new species, ground beetle, cavernicolous, Jiangxi, China

## Abstract

*Morimotoidius zhushandong* **sp. n.** is described and illustrated from a limestone cave called Zhushan Dong II in Wanzhai Xian (=County) of western Jiangxi Province, eastern China. This species is the most modified species within the tribe Platynini in China by having very slender body and appendages, extremely elongated head, and especially, narrowed and barrel-liked pronotum which is as wide as head. However, the above derived characters are autapomorphies to adapt the subterranean environment. *M. zhushandong* **sp. n.** must be a troglobite though it has well pigmented body and flat eyes.

## Introduction

Carabidae is the largest family in the suborder Adephaga of Coleoptera, containing more than 34000 species (subspecies) in the world (Lorenz 2005). They are living in various habitats including in caves. To adapt subterranean environment, the ground beetles generally have several modified morphological characters, such as elongate body and appendages, long and very developed sensory setae, reduced or totally disappeared eyes, and more or less depigmented body (Casale et al. 1998, Faille et al. 2010). Cave-dwelling ground beetles have been reported from over twenty tribes worldwide (Casale et al. 1998), but only four tribes, *viz.*, Tachyini, Trechini, Platynini and Clivinini, recorded in China (Tian 2008, 2013; Deuve and Tian 2011).

In general, species diversity of cavernicolous platynines is much less than that of trechines. For example, about 90 species of trechines in over 30 genera have been reported in China, in contrast to 13 species of platynine in two genera: 12 species in *Jujiroa* Uéno, 1952 (Jedlička 1961; Vigna Taglianti 1995; Uéno and Kishimoto 2001; Deuve 2004; Terada et al. 2005; Uéno 2007), and one in *Xestagonum* Habu, 1978 (Deuve 2001).

During a subterranean biological survey of last year in western Jiangxi Province, a peculiar platynine species was discovered in a limestone cave. On the basis of its pronotal and leg characters, it belongs to the genus *Morimotoidius* Habu, 1954, but represents a lineage which is much different from other congeners. This species is the first record of *Morimotoidius* in mainland China. It is also one of the most modified species of Platynini in the world.

## Materials and methods

All specimens for this study are composed of sixteen specimens. They were collected by hands on walls and ceilings in a limestone cave called Zhushan Dong II in eastern Jiangxi Province, and kept in 50% ethanol before study. Dissections were made by using standard techniques. Body was prepared on paper card; pieces of buccal appendages or genital organs were put on small paper cards and then pinned beneath the specimens from which they were removed. Observation and dissections were made under Leica S8AP0 stereo-binocular microscope. Female genitalia were dipped 10% KOH for one day before dissection, then cleaned in lactic acid for one day, and stained in Chlorazol Black dissolved in 70% ethanol for thirty seconds. Digital photographs were taken by a Canon EOS 40D camera, and then processed by using Adobe Photoshop CS5 computer software.

Body length was measured from apex of right mandible (in opened position) to apex of elytra; body width (=width of elytra) was the maximum distance across elytra.

Abbreviations of measurements used in the text are as following:

HL head length, linear distance from apical margin of right mandible (in opened position) to the occipital suture

HW head width, maximum distance across head, including eyes

PL length of pronotum, distance measured from front to basal margins, along midline

PW width of pronotum, maximum distance across pronotum, along the widest point

PWA width of pronotum at apex, linear transverse distance along front margin

PWB width of pronotum at base, linear transverse distance between hind angles

EL length of elytra, measured from base of scutellum to apex of elytra, through suture

EW width of elytra

Terminology for female reproductive tract follows Deuve (1993) and Liebherr and Will (1998).

## Taxonomic treatment

### Morimotoidius zhushandong

Pang & Tian sp. n.

http://zoobank.org/96870F47-6129-474B-AB13-677EDD9D12C4

http://species-id.net/wiki/Morimotoidius_zhushandong

**Figure 1.**
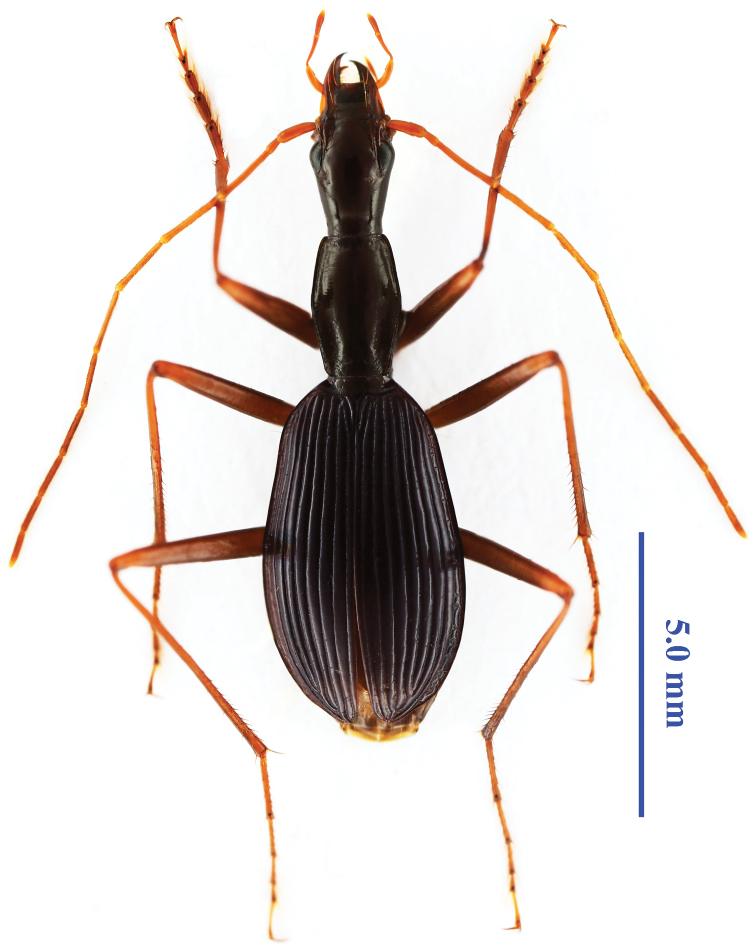
*Morimotoidius zhushandong* sp. n., habitus of male, paratype.

Figs 1 [Fig F2] [Fig F3] [Fig F4] [Fig F5] –19

#### Description.

Length: 11.5–12.5 mm; width: 3.6–3.7 mm. Habitus as in Figure 1. Body extremely slender and elongate, with very long antennae, legs and mouthpart palps.

Black, but ventral surface, femora (except basal and apical tips), clypeus, labrum and apical half of mandibles dark brown, legs including basal and apical tips of femora, antennae, palps, and basal half of mandibles yellow to yellow brown; elytra with indistinct purplish metallic sheen.

Macrosculpture: Strongly shining, surface glabrous, polish and smooth, but base of pronotum, mesosternum, meso- and metepisterna coarsely and sparsely punctate.

Microsculpture: Engraved meshes moderately transverse on labrum, clypeus and base of frons, and base of pronotum; strongly transverse on head; striate on disc of pronotum and elytra, but clearly isodiametric on scutellum.

Head very long and narrow, strongly elongated, much longer than wide, HL/HW = 2.04–2.15 (mean 2.11); widest at level of eyes, and gradually narrowed backwards to neck constriction which is short but distinct, tempora almost straight but slightly curved just before neck constriction; eyes rather flat, more or less depressed; ventral margins of eye well separated from buccal fissure; supraorbital areas with two pairs of setiferous pores, anterior closer to margin of eye than posterior; posterior pore at about middle of head from clypeal suture to neck; interspaces between anterior pores distinctly wider than that between posterior ones; distance between anterior and posterior pores slightly longer than diameter of eye; distance between eye to buccal fissure distinctly shorter than that between posterior pore to eye, but slightly longer than that between anterior pore to eye; frontal impressions rather shallow and wide, ending before anterior supraorbital pores; frons and vertex moderately convex; clypeus moderately transverse, bisetose, labrum subquadrate, almost straight at front, sexsetose; mandibles elongate, gently and gradually narrowed towards apex, apical teeth slightly hooked; right mandible with a small but distinct anterior retinacular tooth at about median portion which is far from other teeth; both right and left mandibles with short and blunt terebral and posterior retinacular teeth near base; ligula broad, bisetose at apex; labial suture well developed; mentum bisetose, apical margin biconcave, with a long and simple median tooth which is widened at apex, epilobes evenly rounded; submentum bearing two pairs of setae, inner ones much long the outer; palps long and slender, subcylindrical, glabrous and asetose except for labial palpomere 2 which is bisetose on inner margin; labial palpomere 2 distinctly longer than 3; maxillary palpomere 3 slightly longer than 4. Antennae long and slender, filiform, extending at about apical 1/5 of elytra in female, but 1/6 in male; antennomeres 1–3 glabrous, antennomere 1 with a long subapical seta, 2 with a short subapical seta, 3 with several apical setae; finely pubescent from antennomere 4, each of 4 to 11 with several apical setae; antennomere 2 the shortest, half as long as 1, antennomeres 3–5 longer than other, each about twice as long as 1; gradually shortened from antennomeres 6 to 11, antennomere 11 almost as long as 1.

Pronotum narrow and strongly elongate, barrel-like, distinctly shorter than head, almost as wide as head including eyes; much longer than wide, PL/PW = 1.59–1.62 (mean 1.60) in male, 1.40–1.43 (mean 1.41) in female; front slightly narrower than base, PWB/PWA = 1.11–1.14 (mean 1.13) in male, 1.09–1.12 (mean 1.10) in female; widest at about middle, gently narrowed towards both fore and hind angles; front and base finely bordered, lateral margins unbordered, but with evenly and distinctly explanate-reflexed areas throughout, marginal setae absent; basal foveae wide and long; fore angle nearly rectangular, hind angle broad though somewhat rectangular; both front and base almost straight; median line deep and long; disc slightly convex, basal area rather flat, with lateral areas of basal parts evidently depressed; propleura faintly tumid, faintly visible from above, at least at the widest part; prosternal process unbordered at apex; scutellum moderately sized.

Elytra very slender, elongate-ovate, much wider than head and pronotum; well bordered at base, base small, shoulders indistinct; widest at a little behind middle, EL/EW = 1.62–1.78 (mean 1.74), more contracted towards apices than towards base; disc moderately convex though rather flat in basal 1/4; striae very deep, continuous and smooth, weakly punctured; intervals strongly convex, stria 3 with three dorsal setiferous pores, basal one close to stria 3, both middle and subapical ones close to stria 2; other intervals without pore; subapical sinuation rather straight, apex broad; preapical and two apical pores present; marginal series of umbilicate pores not aggregated, which are composed of about nineteen pores, denser in subhumeral and subapical areas, sparser in middle portion; three pores (one at subhumerus, other two at subapical area) bearing much longer setae than others, which is distinctly longer than metatarsomere 4; scutellar pores present; scutellar striae deep and rather long. Hind wings reduced.

**Figures 2–10. F2:**
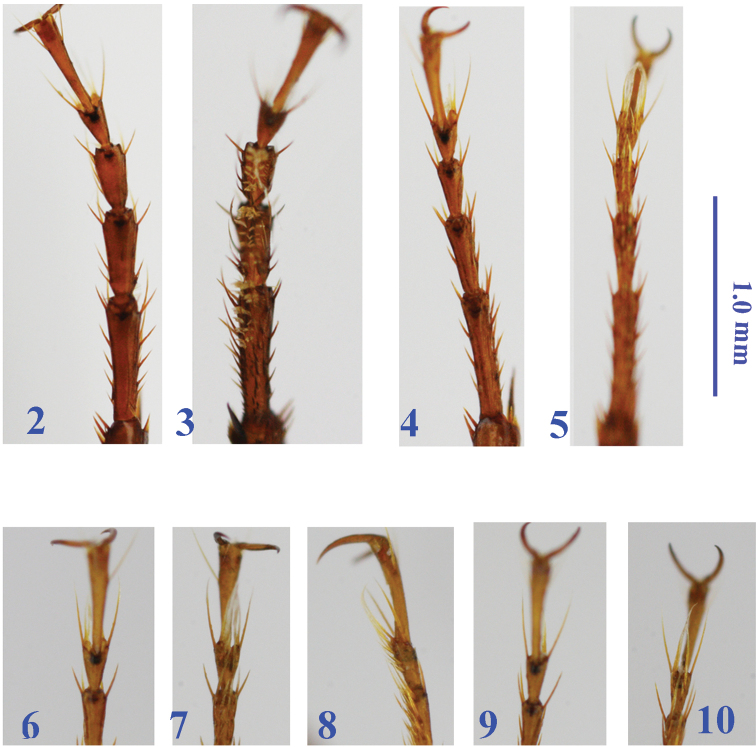
Tarsi of *Morimotoidius zhushandong* sp. n., **2** protarsi, dorsal view, male **3** protarsi, ventral view, male **4** protarsi, dorsal view, female **5** protarsi, ventral view, female **6** mesotarsomeres 3–5, dorsal view, male **7** mesotarsomeres 3–5, ventral view, male **8** mesotarsomeres 3–5, lateral view, male **9** metatasomeres 3–5, dorsal view, male **10** metatasomeres 3–5, ventral view, male.

Legs very long and slender; fore leg short and stout (Figs 2–5); procoxa asetose, meso- and metacoxae bisetose, inner seta of metacoxa absent; trochanters unisetose; femora very slender, profemora unisetose ventrally, meso- and metafemora with three and two ventral setae respectively; tibiae and tarsomeres 1–3 longitudinal bisulcute dorsally; protarsomeres 1–3 slightly dilated in male, with two rows of short and sparse spongy setae ventrally (Fig. 3), while much narrower and without spongy setae in female (Fig. 5); protarsomeres 4 shortly but distinctly emarginate at apex, with lobes nearly symmetric, each with a row of three setae ventrally; meso- and metatarsomeres 4 without subapical setae (Figs 6–10); tarsomeres 5 glabrous ventrally; claws smooth.

Each of abdominal ventrite IV–VI with pair of paramedian setae in both sexes; ventrite VII with two pairs of marginal setae in female, but only one pair in male.

**Figures 11–13. F3:**
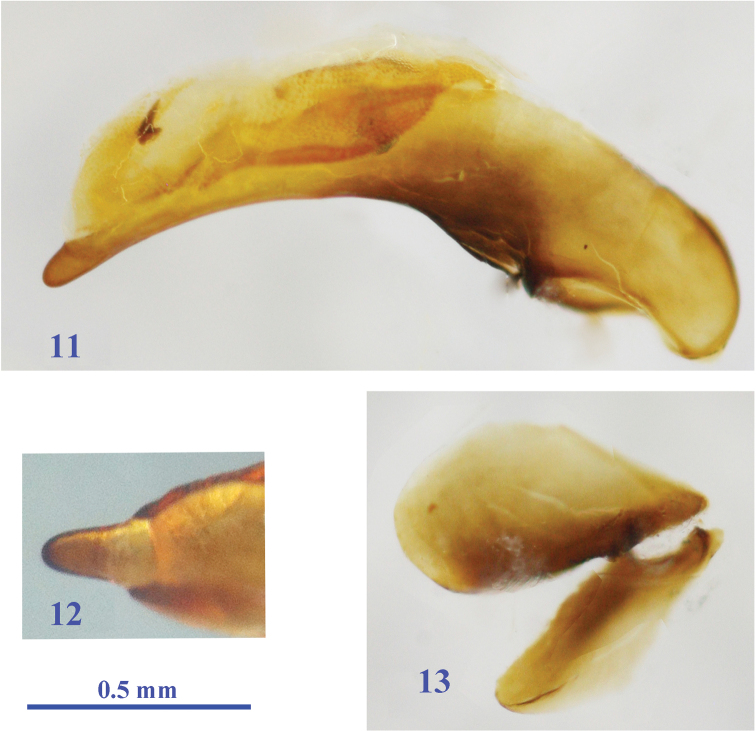
Male genitalia of *Morimotoidius zhushandong* sp. n., **11** median lobe, lateral view **12** apex of median lobe, dorsal view **13** parameres.

Male genitalia (Figs 11–13): The median lobe of aedeagus rather stout, basal bulb large, strongly arcuate in middle portion in lateral view, gently and gradually narrowed towards apex, blunt at tip; dorsal opening wide, nearly as half as whole length, reaching 1/3 from base; apical lamella rather long, nearly twice as long as wide, not parallel-sided, rounded at apex; internal sac with a long copulatory piece covered with scales, and a strongly sclerotized spine dorsally; left paramere styloid, not elongated, smaller and shorter than the right.

**Figure 14. F4:**
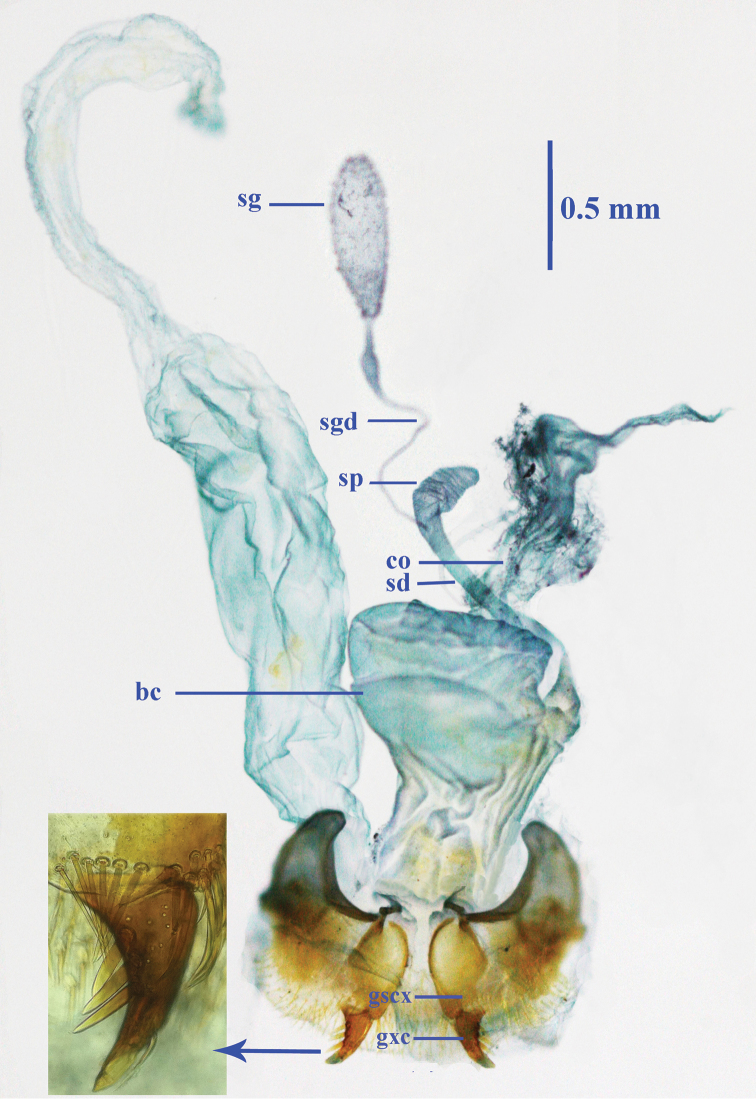
Female reproductive tract of *Morimotoidius zhushandong* sp. n., **bc** bursa copulatrix; **co** common oviduct; **gcx** gonocoxite; **gscx** gonosubcoxite; **sd** spermathecal duct; **sg** spermathecal gland; **sgd** spermathecal gland duct; **sp** spermatheca.

Female reproductive tract (Fig. 14): Ventrite X sparsely setose; gonosubcoxite bearing about a dozen fringe setae along apical area, gonocoxite strongly curved, sharp at apex, bearing three lateral and one dorsal ensiform setae; bursa copulatrix wide, with middle part evidently folded, basally narrower; spermathecal gland very large, elongate ovate; spermathecal gland duct thin and long, connected below base of spermathecal reservoir, which is shorter than spermathecal duct.

#### Sexual dimorphisms.

Apart from protarsomeres 1–3 and ventrite VII, sexual differences are also shown on antennae and pronotum: a little longer or more elongate in male than in female.

#### Variability.

In general, maxillary palpomere 3 distinctly longer than 4, but reverse in a male specimen which left maxilla with palpomere 3 shorter than 4.

#### Remarks.

Within platynines, the chaetotaxy on pronotum and meso- and metatarsi are important characters (Habu 1978; Liebherr 1991; Schmidt 2001; Liebherr and Schmidt 2004). We treat this peculiar species a member of the genus *Morimotoidius* Habu, 1954 due to the fact that it shares the following combined generic characters with other species of this genus: pronotal setae absent, submentum quadrisetose, meso- and metatarsomeres 4 without subapical setae, meso- and metafemora bearing two or three setae, and tarsomeres 5 glabrous ventrally. Certainly, other remarkably modified characters such as the very elongate and rhadinoid body shape, the slender and markedly porrected mandibles, the very thin palps, reduced eyes, the markedly prolonged temples and the very small elytral base must be autapomorphies to adapt the subterranean habitat. *Morimotoidius zhushandong* sp. n. is the first member of *Morimotoidius* found in mainland China. Other species of the genus are living either in Japan (two species) or in Taiwan Province of China (one species) (Habu 1954, 1978; Terada et al. 2005).

#### Etymology.

This new species is named after its type locality. In Chinese, “Zhushan” means a mountain or hill which is covered with bamboo forest, “Dong” means cave.

#### Materials examined.

Holotype: male, Zhushan Dong II, Dongkou Cun, Mabu Xiang, Wanzhai Xian, western Jiangxi, China, 28°02'880"N, 114°22'216"E, 142 m, 2–X-2012, Mingyi Tian & Jingli Cheng leg. in the insect collections of South China Agricultural University, Guangzhou, China (SCAU); paratypes: 6 males and 9 females, ibid. in SCAU, except one male and one female in Muséum National d’Histoire Naturelle, Paris, France, and one male and one female in Coll. J. Schmidt (Admannshagen, Germany), respectively.

#### Distribution.

**Figure 15. F5:**
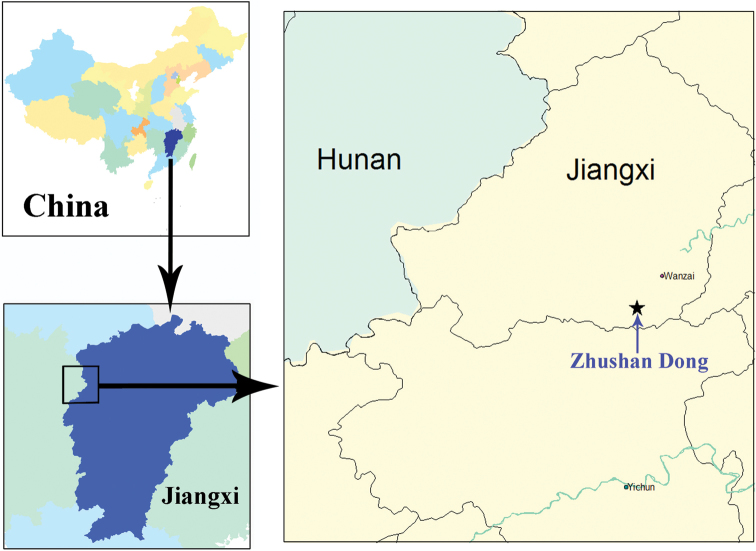
Distribution map of *Morimotoidius zhushandong* sp. n.

China (Jiangxi) (Fig. 15).

#### Habits.

There is unknown about the biology and ecology of *Morimotoidius zhushandong* sp. n. The beetles move quickly on walls and ceilings, feeding on other small arthropods, probably including eggs of the crickets which are common in the cave. The extremely modified troglomorphic characters mentioned above reveal that this beetle has ability to adapt underground environment and probably a troglobite though it has reduced eyes and pigmented body.

#### About the locality cave of *Morimotoidius zhushandong* sp. n.

**Figures 16–19.**
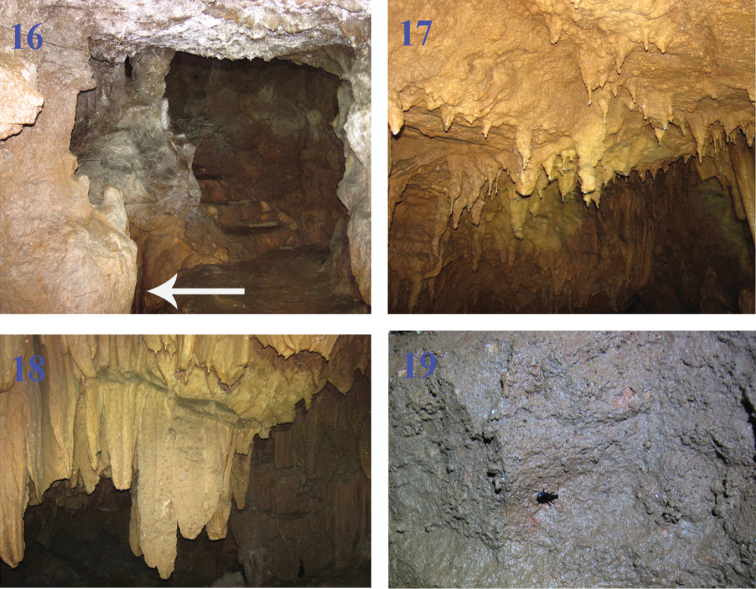
Zhushan Dong II. **16** entrance **17–18** cave walls where the type specimens were collected **19** an adult of *Morimotoidius zhushandong* sp. n. running on cave ceiling.

Zhushan Dong is a touristic cave in western Jiangxi Province, located at Dongkou Cun, Mabu Xiang, Wanzai Xian, not far from the border between Wanzhai Xian and Yichun Shi (=City) (Fig. 15). Actually, there are two limestone caves in the Zhushan Dong scenic areas. Zhushan Dong I is a well-developed touristic cave, as long as 3985 m, with an underground river throughout the main passage. Zhushan Dong II is about 50 meters far from Zhushan Dong I. It is a small cave, about 30 m in length, with a small streamlet moving out at about 5 m inside of the entrance (Fig. 16, indicated by arrowhead). It is still closed for visitors. The beetles were collected by hands on walls and ceilings of the cave (Figs 17–19).

### Key to species of the genus *Morimotoidius* Habu (modified from Habu 1954)

**Table:** 

1	Body slender, head and pronotum extremely elongate, pronotum narrow, barrel-like, as wide as head, interval 3 of elytra with three setiferous pores (Jiangxi Province)	*Morimotoidius zhushandong* sp. n.
–	Body stout, head and pronotum moderate for *Colpodes*, not very elongate, pronotum much wider than head, interval 3 of elytra without setiferous pores	2
2	Tarsomere 5 ciliate ventrally (Taiwan Province)	*Morimotoidius formosus* Habu, 1954
–	Tarsomere 5 glabrous ventrally	Japanese species
	(To separate the two Japanese species, see Habu 1978 for detail)	

## Supplementary Material

XML Treatment for Morimotoidius zhushandong
